# Supplementary material for: A Porcine DNMT1 Variant: Molecular Cloning and Generation of Specific Polyclonal Antibody
Source: Genes (Basel). 2023 Jun 23;14(7):1324. doi: 10.3390/genes14071324 (PMC10379332; doi:10.3390/genes14071324)
Supplement: Supplementary file 1 [file genes-14-01324-s001.zip › Table S2.pdf]

Table S2: The GenBank accession number of predicted porcine DNMT1s.

Name	GenBank Accession Number (mRNA)	GenBank Accession Number (protein)
Porcine DNMT1 X1	XM_005654828.3	XP_005654885.1
Porcine DNMT1 X2	XM_005654829.3	XP_005654886.1
Porcine DNMT1 X3	XM_021082029.1	XP_020937688.1
Porcine DNMT1 X4	XM_021082032.1	XP_020937691.1
Porcine DNMT1 X5	XM_021082036.1	XP_020937695.1
Porcine DNMT1 X6	XM_021082040.1	XP_020937699.1
Porcine DNMT1 X7	XM_021082053.1	XP_020937712.1
Porcine DNMT1 X8	XM_021082064.1	XP_020937723.1
