# Supplementary figures and images for: A Porcine DNMT1 Variant: Molecular Cloning and Generation of Specific Polyclonal Antibody
Source: Genes (Basel). 2023 Jun 23;14(7):1324. doi: 10.3390/genes14071324 (PMC10379332; doi:10.3390/genes14071324)

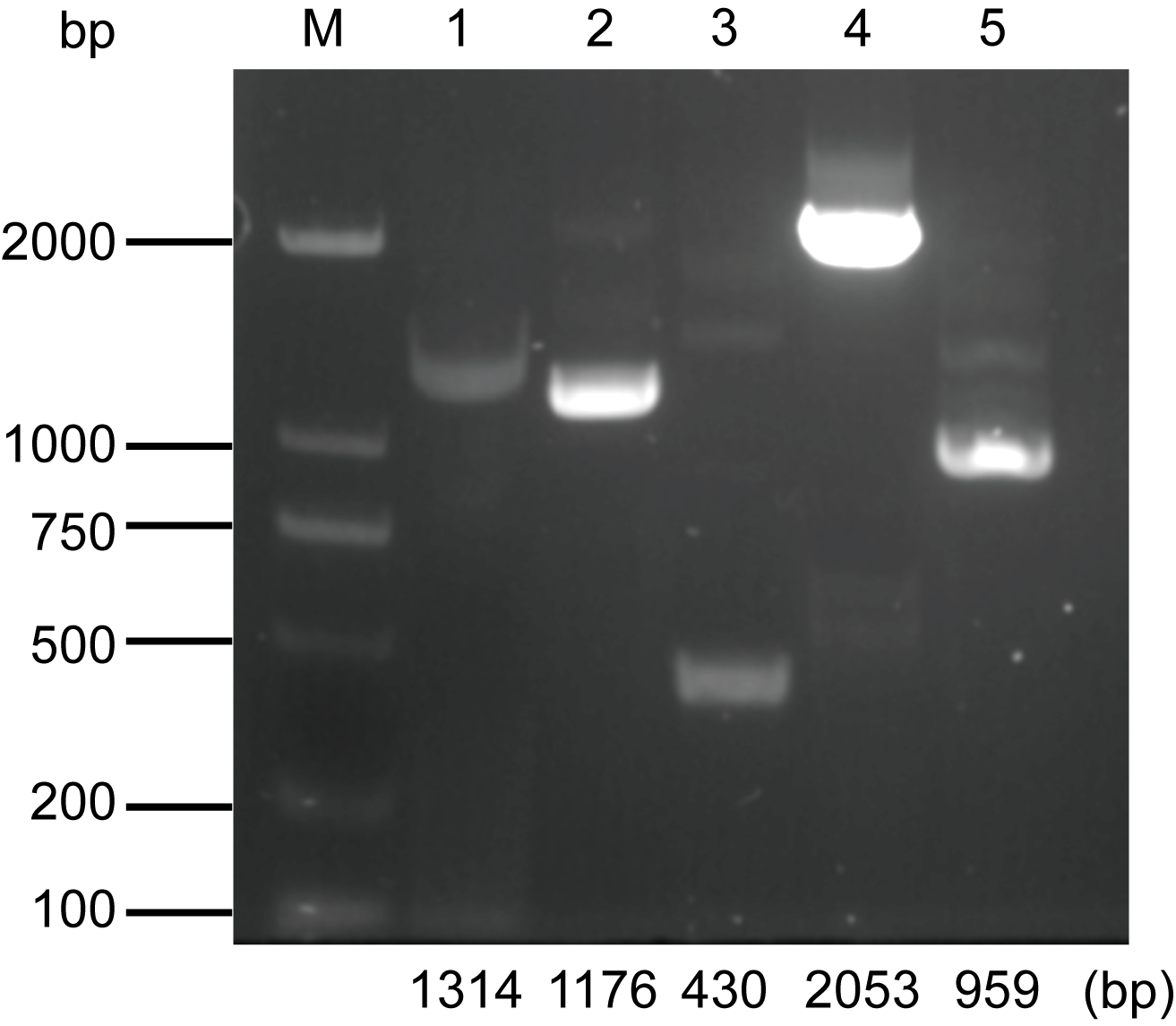

Supplement: Supplementary file 1 [file genes-14-01324-s001.zip › Figure S1.tif]
